# Supplementary figures and images for: Epidemiology of Mosquito-Borne Viruses in Egypt: A Systematic Review
Source: Viruses. 2022 Jul 20;14(7):1577. doi: 10.3390/v14071577 (PMC9322113; doi:10.3390/v14071577)

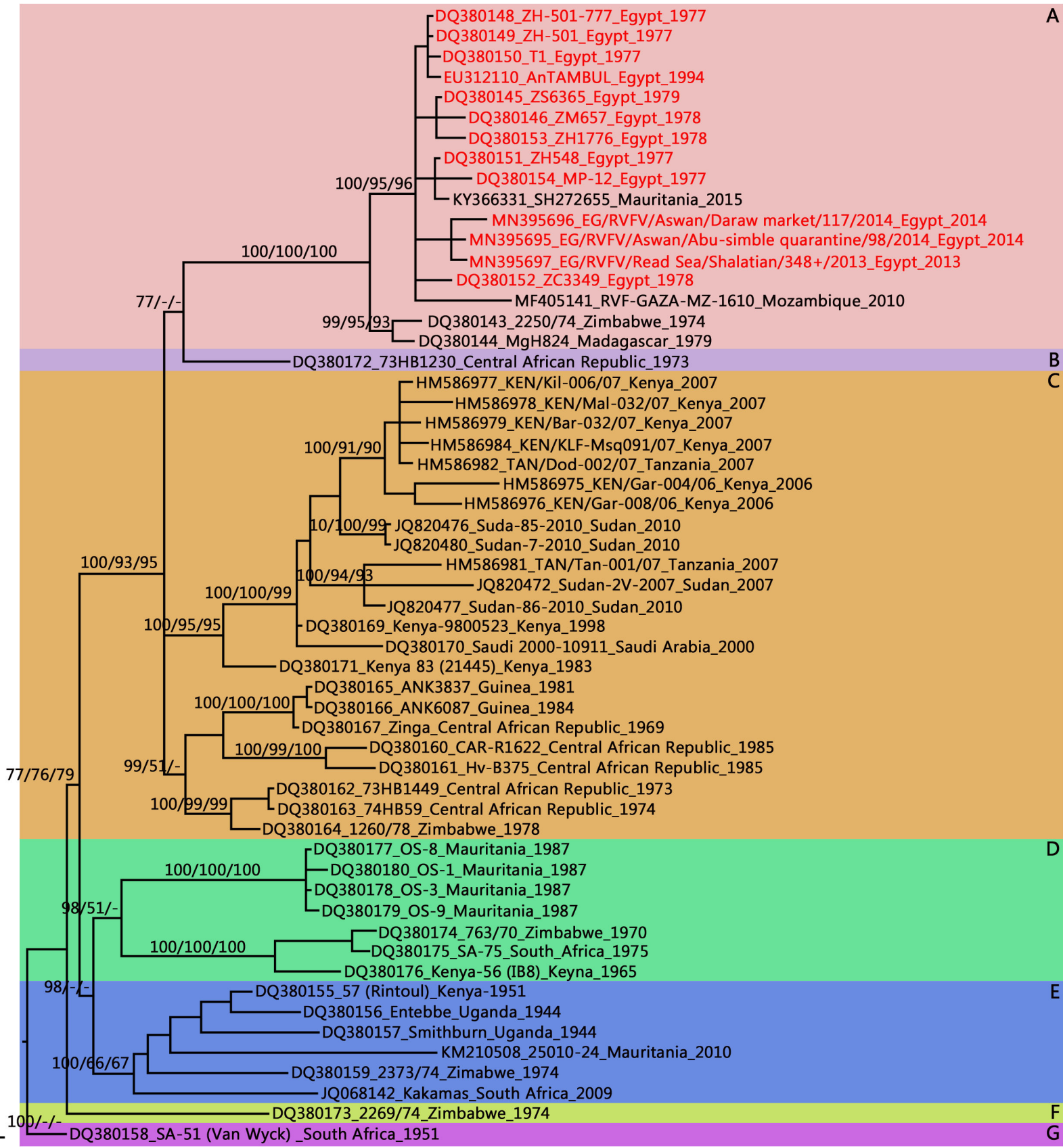

Supplement: Supplementary file 1 [file viruses-14-01577-s001.zip › viruses-1806176-supplementary.pdf]
